# Handling errors in the response: Considerations for leveraging unsupervised or incomplete data for genetic evaluations

**DOI:** 10.3168/jdsc.2024-0668

**Published:** 2025-06-09

**Authors:** Xiao-Lin Wu, John B. Cole, Andres Legarra, Kristen L. Parker Gaddis, João W. Dürr

**Affiliations:** 1 Council on Dairy Cattle Breeding, Bowie, MD 20716; 2 Department of Animal and Dairy Sciences, University of Wisconsin, Madison, WI 53706; 3 Department of Animal Sciences, Donald Henry Barron Reproductive and Perinatal Biology Research Program, and the Genetics Institute, University of Florida, Gainesville, FL 32608; 4 Department of Animal Science, North Carolina State University, Raleigh, NC 27607; 5 Department of Animal and Dairy Science, University of Georgia, Athens, GA 30602

## Abstract

Accurate genetic evaluations rely on high-quality phenotypic data; however, measurement errors and data inconsistencies—such as those arising from unsupervised or incomplete sources—pose challenges to their reliability. This study investigates the effect of response errors on genetic evaluations across continuous and categorical traits. We introduce an additive measurement error model to illustrate how phenotypic errors influence genetic effects and variance estimation. Next, we examine a binary trait scenario, demonstrating the utility of sensitivity and specificity in adjusting observed incidence rates for misclassified data. To further illustrate genetic evaluation in the presence of misclassifications, we proposed a mixed effects liability model assuming unequal sensitivity and specificity or varied false-positive and false-negative rates. Our findings underscore the necessity of integrating measurement error models into genetic evaluation frameworks to reduce bias and enhance predictive accuracy.

## Highlights

• Errors in the response are explained in the context of mixed effects models.
• Linear calibration is demonstrated and applied to calibrate estimated test-day yields.
• Misclassification and reclassification probabilities are defined for a binary trait.
• Sensitivity and specificity are demonstrated when calibrating an unofficial test.
• A liability threshold model accounting for heterogeneous misclassifications is described.

## Graphical Abstract

**Figure:**
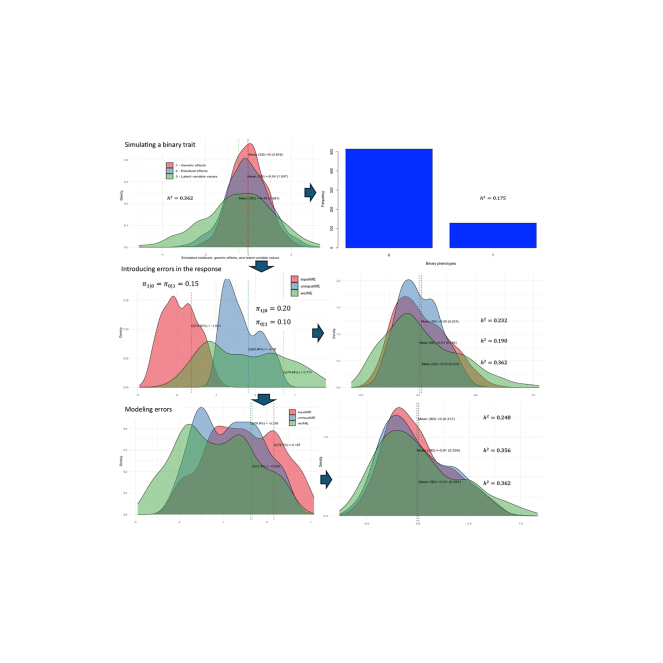
**Summary:** Phenotypic errors stemming from various sources, such as measurement inaccuracies or inconsistencies, introduce noise and bias into genetic analyses, undermining the precision of genetic parameter estimates and selection decisions. This article provides an overview of phenotypic errors and their impacts on genetic evaluations in both continuous and categorical traits. We begin by defining errors in response in the context of phenotypic measurements and discussing their types and sources. The additive measurement error model is introduced for continuous traits to illustrate how phenotypic errors influence the estimation of model effects and variances, lowering the heritability and accuracy of genetic predictions. For categorical traits, we show the utility of sensitivity and specificity in evaluating data quality, leveraging internal validation datasets to calibrate unofficial tests. equalME = a threshold model assuming equal misclassification rates; unequalME = a threshold model assuming unequal misclassification rates; w0/ME = a threshold model fitted on the data with zero misclassifications.

The emergence of high-throughput phenotyping and unofficial, unsupervised data sources has recently transformed phenotypic data landscapes, offering opportunities to accelerate genetic progress and expand trait evaluations. However, these developments introduce significant challenges to data reliability and consistency. Despite these concerns, there is a growing push toward utilizing unsupervised datasets for developing new traits, such as those from massive recording programs or wearable sensors (Heringstad and Wethal, 2023), and automatic phenotyping of hoof health (Siachos et al., 2024) and milking speed (O'Connell et al., 2024). Use of milk Fourier transform mid-infrared spectrometry is also expanding, offering new tools for assessing milk's nutritional quality and technological properties (Soyeurt et al., 2023). However, unsupervised data lack the same quality controls as official testing protocols, increasing the risk of phenotyping errors that can compromise genetic evaluations and breeding outcomes.

This article provides a technical overview of modeling phenotypic errors in continuous and categorical traits. We begin by defining errors in the response in the context of genetic evaluation and discussing their types and sources. An additive measurement error model for a continuous trait is introduced to illustrate how phenotypic errors influence the estimation of additive genetic effects and subsequent variance components. For a binary trait, we show the utility of sensitivity and specificity in assessing data quality, leveraging internal validation to adjust observed incidence rates. A hypothetical example demonstrates the calibration process for an unofficial test with significant misclassifications and the subsequent adjustment of the observed incidence rate.

Consider the following mixed effects model without measurement errors:

[1] y=Xb+Zu+e.

Here, **y** is a vector of the unobserved response variable, **b** is a vector of fixed effects, $u˜\left(0,G\right)$ is a vector of random effects, where $G=A\sigma_{u}^{2}$ is the additive genetic variance-covariance matrix, **A** is the numerator additive genetic relationship matrix, and $\sigma_{u}^{2}$ is the common additive genetic effect variance, **X** and **Z** are the corresponding incidence matrices, and $e˜\left(0,R\right)$ is a vector of residuals, where $R=I\sigma_{e}^{2}$ is the residual variance-covariance matrix, **I** is an identity matrix, and $\sigma_{e}^{2}$ is the common residual variance. The residual covariances are assumed to be nonexistent.

Now, instead of observing **y**, we observe a noisy version, y*, due to measurement errors:

[2] $$y^{*}=y+=\mathrm{Xb}+\mathrm{Zu}+\left(e+\right),$$

where $˜\left(0,I\sigma^{2}\right)$ are independent measurement errors with a common variance $\sigma^{2}.$ Assume $E\left(y^{*}|y\right)=y,$ meaning y* is unbiased for the unobserved **y**. Then, the observed response follows:

[3] $$y^{*}˜\left(\mathrm{Xb},{\mathrm{ZGZ}}^{'}+R+I\sigma^{2}\right).$$

Thus, the additional noise inflates the variance structure of the response, affecting the estimations of fixed and random effects.

Without adjusting the measurement errors, the BLUP estimate of **u** becomes

[4] u^*=GZ'ZGZ'+R+Iσ2-1y*-Xb=GZ'ZGZ'+R+Iσ2-1Zu+e+=GZ'ZGZ'+R+Iσ2-1Zu+e+GZ'ZGZ'+R+Iσ2-1.

In the above, $\mathrm{GZ}‘{\left({\mathrm{ZGZ}}^{‘}+R+I\sigma^{2}\right)}^{-1}$ introduces additional variability attributable to measurement errors, making it less precise.

This assumption about measurement errors is typical of the classic measurement error model, which assumes the distribution of observed phenotypes given the true values, $p\left(y^{*}|y\right).$ An alternative is the Berkson measurement error model, which instead describes the distribution of true phenotypes given the observed values, $p\left(y|y^{*}\right)$ (Buonaccorsi, 2010). In reality, the effects of phenotypic errors can be more complex, and various alternative measurement error models are worth considering. For instance, let $E\left(y^{*}|y,\alpha \right)=1\alpha +y,$ where *α* > 0 is a constant, representing a systematic bias in observed responses. A linear error model, $E\left(y^{*}|y,\alpha ,\beta \right)=1\alpha +\beta y,$ assumes a linear relationship between the observed and the true value, where *α* is the intercept and *β* is the regression coefficient. Still, nonlinear error models, denoted by $E\left(y^{*}|y\right)=g\left(y;H\right),$ introduce nonlinear measurement errors, where *H* collectively represents all hyperparameters.

Consider the following linear measurement error model:

[5] $$y^{*}|y=1\alpha +\beta y+\;.$$

Substituting **y** with Equation [1] gives

[6] $$\begin{array}{c} y^{*}=1\alpha +\beta \left(\mathrm{Xb}+\mathrm{Zu}+e\right)+=\left(1\alpha +\beta \mathrm{Xb}\right)+\beta \mathrm{Zu}+\left(\beta e+\right)\\ =X^{*}b^{*}+Zu^{*}+e^{*}. \end{array}$$

Here, $X^{*}=\left(1,X\right),$ $b^{*}=\left(\begin{array}{c} \alpha \\ \beta b \end{array}\right),$ $u^{*}=\beta u,$ and $e^{*}=\beta e+.$ The observed variances for random effects and residuals (errors) are $\sigma_{u^{*}}^{2}=\beta^{2}\sigma_{u}^{2}$ and $\sigma_{e^{*}}^{2}=\beta^{2}\sigma_{e}^{2}+\sigma^{2}.$ The fixed and random effects can be obtained by solving the following mixed effects model equations:

[7] X*'R*-1X*X*'R*-1ZZ'R*-1X*Z'R*-1Z+G*-1b^*u^*=X*'R-1yZ'R-1y,

where $G^{*}=A\beta^{2}\sigma_{u}^{2}$ and $R^{*}=I\left(\beta^{2}\sigma_{e}^{2}+\sigma^{2}\right).$

Then, the estimated fixed and random effects accounting for phenotypic errors are

[8] $$\hat{b}=\frac{1}{\hat{\beta }}{\left(X'X\right)}^{-1}\left(X'X^{*}{\hat{b}}^{*}-X'1\hat{\alpha }\right),$$

[9] $$\hat{u}=\frac{1}{\hat{\beta }}{\hat{u}}^{*}.$$

The variance components accounting for phenotypic errors are

[10] $${\hat{\sigma }}_{u}^{2}=\frac{1}{{\hat{\beta }}^{2}}{\hat{\sigma }}_{u^{*}}^{2},$$

[11] $${\hat{\sigma }}_{e}^{2}=\frac{1}{{\hat{\beta }}^{2}}\left({\hat{\sigma }}_{e^{*}}^{2}-{\hat{\sigma }}^{2}\right).$$

The above illustrates the principle of linear error model calibration. Calibration typically requires an internal or external sample to assess the relationship between the measured values (prone to error) and the true values of the response variable. To demonstrate this approach, we apply it to mitigate measurement errors in estimated daily milk yields for demonstration. The dataset consists of 15,888 Holstein milking records from 3,717 animals, randomly sampled from 23 herds across 11 US states, covering the first 3 lactations between 2006 and 2009 (Wu et al., 2023). Daily milk yields were calculated from partial (AM or PM) yields using the DeLorenzo-Wiggans (**D-W**; DeLorenzo and Wiggans, 1986) model. The dataset was randomly divided into 3 equal portions based on unique animal ID. Two-thirds were used to fit the calibration equation, whereas the remaining one-third was used to validate the calibration model.

The linear calibration equations are presented in Table 1. The D-W model tended to inflate the variance of estimated daily milk yields. The ratio of estimated to actual daily milk yield variances ranged from 1.04 to 1.17 for the morning milkings and 1.06 to 1.30 for the evening milkings. The calibration equations varied across lactation months. Applying these linear calibrations increased the accuracy of estimated daily milk yields by approximately 1%.

**Table 1:** Linear calibration regression for the daily milk yields estimated using the DeLorenzo and Wiggans (1986) model^1^

Month in milk	Morning milkings						Evening milkings					
	$\sigma_{y}^{2}$	$\sigma_{y^{*}}^{2}$	*K*	*a*	*b*	$\sigma^{2}$	$\sigma_{y}^{2}$	$\sigma_{y^{*}}^{2}$	*K*	*a*	*b*	$\sigma^{2}$
1	111.2	119.3	1.07	0.238	0.979	12.6	111.2	125.8	1.13	−0.133	1.003	13.9
2	99.8	103.6	1.04	1.234	0.961	11.5	99.8	113.2	1.13	−0.499	1.004	12.5
3	82.6	90.7	1.10	0.110	0.986	10.4	82.6	90.0	1.09	0.640	0.980	10.6
4	69.0	75.2	1.09	2.488	0.936	14.7	69.0	89.7	1.30	−1.843	1.032	16.2
5	62.8	69.1	1.10	1.682	0.955	11.8	62.8	77.7	1.24	−1.242	1.017	12.7
6	54.0	60.9	1.13	1.343	0.963	10.9	54.0	67.8	1.26	−1.359	1.022	11.5
7	55.3	63.5	1.15	0.767	0.970	11.5	55.3	67.4	1.22	−0.396	1.000	12.1
8	54.9	63.2	1.15	0.272	0.986	9.85	54.9	63.6	1.16	0.003	0.984	10.3
9	52.8	62.0	1.17	−0.568	1.018	7.23	52.8	56.9	1.08	0.720	0.962	8.04
10	59.0	62.5	1.06	0.703	0.965	7.67	59.0	69.0	1.17	−0.515	1.013	8.52
11	62.4	73.1	1.17	−0.658	1.020	8.23	62.4	65.9	1.06	0.659	0.961	8.22
12	67.3	73.1	1.09	0.263	0.978	8.74	67.3	75.6	1.12	−0.015	0.993	9.25

1 $\sigma_{y}^{2}$ = actual daily milk yield (DMY) variance; $\sigma_{y^{*}}^{2}$ = estimated DMY variance; $K=\frac{\sigma_{y^{*}}^{2}}{\sigma_{y}^{2}};$*a*,*b* = intercept and regression coefficient of the linear regression calibration equation; $\sigma^{2}$ = error variance.

For a categorical trait, measurement errors lead to misclassifications, altering the observed phenotypic variance. Consider a binary disease trait, where the phenotype is often coded as *y* = 0 (healthy) or *y* = 1 (sick). Let

[12] $$q^{*}=q+\;\Delta ,$$

where $q^{*}$ is the observed incidence rate, *q* is the unobserved true incidence rate, and Δ denotes the difference between them. Assuming a Bernoulli distribution, the observed variance is

[13] $$\begin{array}{c} Var\left(y^{*}\right)=q^{*}\left(1-q^{*}\right)=\left(q+\Delta \right)\left(1-q-\Delta \right)\\ =q\left(1-q\right)+\left(\Delta -2q\Delta -\Delta^{2}\right). \end{array}$$

Here, y* represents an observed phenotype subject to misclassification. Compared with the true variance $Var\left(y\right)=q\left(1-q\right),$ the observed variance deviates by $\left(\Delta -2q\Delta -\Delta^{2}\right)>0$ and decreases if $\left(\Delta -2q\Delta -\Delta^{2}\right)<0.$

Let $\pi_{y^{*}|y}=p\left(y^{*}|y\right),$ which represents the misclassification probability of y* given *y*. Statistically, sensitivity $\left(\pi_{1|1}\right)$ is the conditional probability of observing a positive case when the true status is positive:

[14] $$\pi_{1|1}=p\left(y^{*}=1|y=1\right).$$

Specificity $\left(\pi_{0|0}\right)$ is the conditional probability of observing a negative case when the true status is negative:

[15] $$\pi_{0|0}=p\left(y^{*}=0|y=0\right).$$

Then, the probability of a false negative $\left(\pi_{0|1}\right)$ is 1 minus the sensitivity of the test:

[16] $$\pi_{0|1}=p\left(y^{*}=0|y=1\right)=1-\pi_{1|1}.$$

The probability of a false positive $\left(\pi_{1|0}\right)$ is 1 minus the specificity:

[17] $$\pi_{1|0}=p\left(y^{*}=1|y=0\right)=1-\pi_{0|0}.$$

A reliable test often aims for sensitivity and specificity of at least 90%. However, in practice, the cutoffs for these 2 measures must balance the risks of false negatives and false positives.

A Berkson error model specifies the distribution of *y* given y*, known as the reclassification probability or predictive probability:

[18] $$\lambda_{y|y^{*}}=p\left(y|y^{*}\right).$$

This probability describes the true phenotype given the observed value. To relate the reclassification probability to the misclassification probability, we have

[19] $$\begin{array}{c} \lambda_{1|1}=p\left(y=1|y^{*}=1\right)=\frac{p\left(y=1,y^{*}=1\right)}{p\left(y^{*}=1\right)}=\\ =\frac{\pi_{1|1}q}{\pi_{1|1}q+\left(1-\pi_{0|0}\right)\left(1-q\right)}, \end{array}$$

[20] $$\begin{array}{c} \lambda_{0|0}=p\left(y=0|y^{*}=0\right)=\frac{p\left(y=0,y^{*}=0\right)}{p\left(y^{*}=0\right)}=\\ =\frac{\pi_{0|0}\left(1-q\right)}{\left(1-\pi_{1|1}\right)q+\pi_{0|0}\left(1-q\right)}, \end{array}$$

where $q=p\left(y=1\right).$ Similarly, we have

[21] $$\lambda_{0|1}=1-\lambda_{1|1},$$

[22] $$\lambda_{1|0}=1-\lambda_{0|0}.$$

When misclassifications occur, a naïve inference estimates the observed incidence as the sample proportion with $y^{*}=1.$ The expected marginal incidence rate is calculated as follows:

[23] $$\begin{array}{c} E\left(q^{*}\right)=p\left(y^{*}=1\right)\\ =\left(1-\pi_{0|0}\right)\left(1-q\right)+\pi_{1|1}q\\ =q\left(\pi_{1|1}+\pi_{0|0}-1\right)+1-\pi_{0|0}. \end{array}$$

Because $E\left(q^{*}\right)\neq q,$ the difference represents the bias in the naïve estimate, ${\hat{q}}^{*},$ as follows:

[24] $$\mathrm{bias}\left({\hat{q}}^{*}\right)=E\left(q^{*}\right)-q=q\left(\pi_{1|1}+\pi_{0|0}-2\right)+1-\pi_{0|0}.$$

Rearranging Equation [23] provides the following adjustment formula:

[25] $$\hat{q}=\frac{{\hat{q}}^{*}-\left(1-{\hat{\pi }}_{0|0}\right)}{{\hat{\pi }}_{1|1}+{\hat{\pi }}_{0|0}-1}.$$

where ${\hat{q}}^{*}=E\left(q^{*}\right),$ empirically bounded between 0 and 1 (Buonaccorsi, 2010). Similarly, by reversing the roles of *y* and y*, we derive an alternative adjustment formula based on reclassification probabilities:

[26] $$\hat{q}={\hat{q}}^{*}\left(\lambda_{1|1}+\lambda_{0|0}-1\right)+1-\lambda_{0|0}.$$

A hypothetical example is represented in Table 2, where 2,580 animals are divided into 2 subsets, representing a double-sampling approach. The first subset, consisting of 980 animals, serves as an internal calibration set, where animals are diagnosed using the official (error-free) and unofficial (error-prone) methods. The second subset included 1,600 animals diagnosed only with the unofficial test. The overall observed incidence rate was ${\hat{q}}^{*}=14.0\&percnt;,$ whereas in the validation set, the incidence rate was 10.2%.

**Table 2:** An illustrative example of double sampling: *y* = true status (official tests without errors) and y* = phenotype subject to misclassification (unofficial tests)

Item		$y^{*}=0$	$y^{*}=1$	Sum
Internal validation	*y* = 0	$n_{00}=840$	$n_{01}=40$	880
	*y* = 1	$n_{10}=3$	$n_{11}=97$	100
Test set	*y* = ?	1,375	225	1,600
Sum		2,218	362	2,580

In the internal validation set, there were 40 false positives among the 880 true-negative cases and 3 false negatives among the 100 true-positive cases. Thus, the sensitivity and specificity are computed as follows: ${\hat{\pi }}_{1|1}=\frac{97}{97+3}=0.970;$ ${\hat{\pi }}_{0|0}=\frac{840}{840+40}=0.955.$ Using Equation [25], the observed incidence rate is adjusted to $\hat{q}=\frac{0.140-\left(1-0.955\right)}{0.970+0.955-1}\times 100\&percnt;=10.3\&percnt;.$ This calibration decreases the incidence rate by 3.7 due to misclassification.

In the double-sampling design, reclassification rates are often used (Buonaccorsi, 2010). The reclassification rates are calculated as ${\hat{\lambda }}_{1|1}=\frac{97}{97+40}=0.708$ and ${\hat{\lambda }}_{0|0}=\frac{840}{840+3}=0.996.$ Then, using Equation [26], the adjusted incidence rate is recalculated as $\hat{q}=\left(0.140\times \left(0.708+0.996-1\right)+1-0.996\right)$ × 100% = 10.3%. Both approaches yield almost identical adjusted incidence rates in this example.

Now, consider the genetic evaluations of a binary trait. Extending the method from a binary trait to a categorical trait is straightforward, involving dealing with multiple thresholds. The threshold model assumes a continuous and normally distributed variable, known as a liability (*η*), that delimits the observable phenotypes by a threshold *τ* (Sorensen and Gianola, 2002):

[27] $$y|\eta ,\tau =\left(\begin{array}{c} \begin{array}{cc} 1 & \;\;\;\mathrm{if}\;\eta >\tau \end{array}\\ \begin{array}{cc} 0 & \mathrm{otherwise}\; \end{array} \end{array}\right).$$

The latent reliability variable *η* is treated as the dependent variable, replacing the observed phenotypes in the mixed effects model [1]. The threshold is computed as follows:

[28] $$\tau =\Phi^{-1}\left(1-\hat{q}\right),$$

where $\hat{q}$ is the incidence rate.

With misclassifications, the probabilities of observed phenotype $y^{*}$ given the latent variable *η* for the true phenotype are defined as follows:

[29] $$p\left(y^{*}=1|y=1\right)=\pi_{1|1}$$

[30] $$p\left(y^{*}=0|y=1\right)=1-\pi_{1|1}$$

[31] $$p\left(y^{*}=1|y=0\right)=1-\pi_{0|0}$$

[32] $$p\left(y^{*}=0|y=0\right)=\pi_{0|0}$$

This liability threshold model is similar to the standard threshold model (Sorensen and Gianola, 2002) except for the likelihood function, which is the following:

[33] $$\begin{array}{c} L\left(b,u|y^{*}\right)\propto P\left(y^{*}|b,u\right)=\prod_{i=1}^{n}\left(\left(y_{i}^{*}|y_{i},\right)P\left(y_{i}|l_{i},t\right)\right)\\ =\prod_{i=1}^{n}{\left(\left(1-\pi_{0|1}\right)\left(1-\Phi \left(\frac{\tau -x_{i}^{'}b-z_{i}^{'}u}{\sigma_{e}^{2}}\right)\right)+\pi_{0|1}\Phi \left(\frac{\tau -x_{i}^{'}b-z_{i}^{'}u}{\sigma_{e}^{2}}\right)\right)}^{y_{i}^{*}}\\ +{\left(\pi_{1|0}\left(1-\Phi \left(\frac{\tau -x_{i}^{'}b-z_{i}^{'}u}{\sigma_{e}^{2}}\right)\right)+\left(1-\pi_{1|0}\right)\Phi \left(\frac{\tau -x_{i}^{'}b-z_{i}^{'}u}{\sigma_{e}^{2}}\right)\right)}^{\left(1-y_{i}^{*}\right)}. \end{array}$$

The above likelihood extends the likelihood function [2] in Rekaya et al. (2001). Our model allows different false-positive and false-negative rates, whereas Rekaya et al. (2001) assumed a common parameter *π* for misclassifications, enforcing equal false positives and false negatives. With a Bayesian implementation via Markov chain Monte Carlo (**MCMC**) simulation, this change requires additionally generating an indicator variable for each animal before sampling reliabilities, where *δ_i_* = 1 if there is a misclassification or 0 otherwise. The asymmetric misclassification rates can be treated as known a priori or unknown to be estimated.

To illustrate how our model works, we simulated a binary disease trait using a mixed effects, animal model for 643 cows derived from a true pedigree consisting of 125 sires and 477 dams (Connor et al., 2013). A single, arbitrary fixed-effect variable with 10 levels was simulated from a standardized normal distribution. The residuals were generated with a multivariate normal distribution: $e˜\left(0,I\sigma_{e}^{2}\right),$ where $\sigma_{e}^{2}$ = 1. Additive genetic values were generated from $u˜\left(0,A\sigma_{u}^{2}\right),$ where $\sigma_{u}^{2}=\frac{h^{2}}{1-h^{2}}\times \sigma_{e}^{2}$ and *h*^2^ = 0.4. The heritability calculated from simulated genetic and residual variances was 0.397, slightly lower than the 0.40 due to Monte Carlo errors. A latent variable was generated as a sum of the fixed, additive genetic, and residual effects. Delimiting the latent variable using the 80th quantile of its cumulative distribution as the threshold (*τ* = 0.865) generated a binary trait with an incidence rate of 20.06%. Discretizing a continuous phenotype (*h*^2^ = 0.362) led to a binary trait with a lower heritability (*h*^2^ = 0.175). See the upper figure of the Graphical Abstract.

Misclassifications were simulated assuming equal (**equalME**) versus unequal (**unequalME**) false-positive and -negative rates. Under equalME, we set $\pi_{1|0}=\pi_{0|1}=0.15,$ equivalent to setting $\pi_{0|0}=\pi_{1|1}=0.85.$ The observed incidence rate was 34.06%. Under unequalME, we set $\pi_{1|0}=0.2$ and $\pi_{0|1}=0.1,$ equivalent to letting $\pi_{0|0}=0.8$ and $\pi_{1|1}=0.9.$ The observed incidence was 29.08%. Further, a baseline model fitted the data without misclassifications (**wo/ME**). Linear and threshold Bayesian models were implemented via MCMC simulation (Sorensen and Gianola, 2002). We ran 100,000 iterations for each analysis, with a 20,000 burn-in, and thinned every tenth iteration.

Introducing errors in the response variable led to decreased heritability estimates. On the observable scale, the estimated heritabilities were 0.175 (wo/ME), 0.148 (equalME), and 0.134 (unequalME), reflecting a relatively greater proportion of residual variance relative to genetic variance in the presence of misclassifications. On the liability scale, the estimated heritabilities were 0.362 (wo/ME), 0.232 (equalME), and 0.190 (unequalME). Unequal error rates led to a relatively greater decrease in the heritability estimates. With the residual variance fixed at 1.00, the estimated genetic variance decreased from 0.569 (wo/ME) to 0.302 (equalME) and 0.235 (unequalME). Under both scenarios, the correlation between the simulated and estimated breeding values decreased from 0.487–0.495 (wo/ME) to 0.336–0.337 (equalME) and 0.334–0.339 (unequalME).

Assuming equal error rates, our model is equivalent to the model proposed by Rekaya et al. (2001), and both approaches produced almost identical results (data not presented). However, our model showed some advantages when the 2 types of error rates varied. A priori, the adjusted incidence rate was 20.1%, close to the simulated value, using Equation [25] assuming unequal sensitivity and specificity $(\pi_{0|0}=0.8$ and $\pi_{1|1}=0.9).$ The posterior incidence estimate was 24.3%, with an estimated heritability of 0.356. In contrast, when assuming equal sensitivity and specificity $(\pi_{0|0}$ = $\pi_{1|1}=0.85),$ the adjusted incidence was higher (23.8%). The posterior incidence was 29.2%, with a heritability of 0.248. The former model also gave a higher correlation between the simulated and estimated breeding values (0.491) than the latter model (0.478). Both correlations were higher than those without accounting for misclassifications (0.337–0.339). Compared to a baseline model without misclassifications, our model, assuming unequal sensitivity and specificity, exhibited higher correlations in estimated liabilities and genetic values than the model assuming equal sensitivity and specificity (Figure 1).

**Figure 1:**
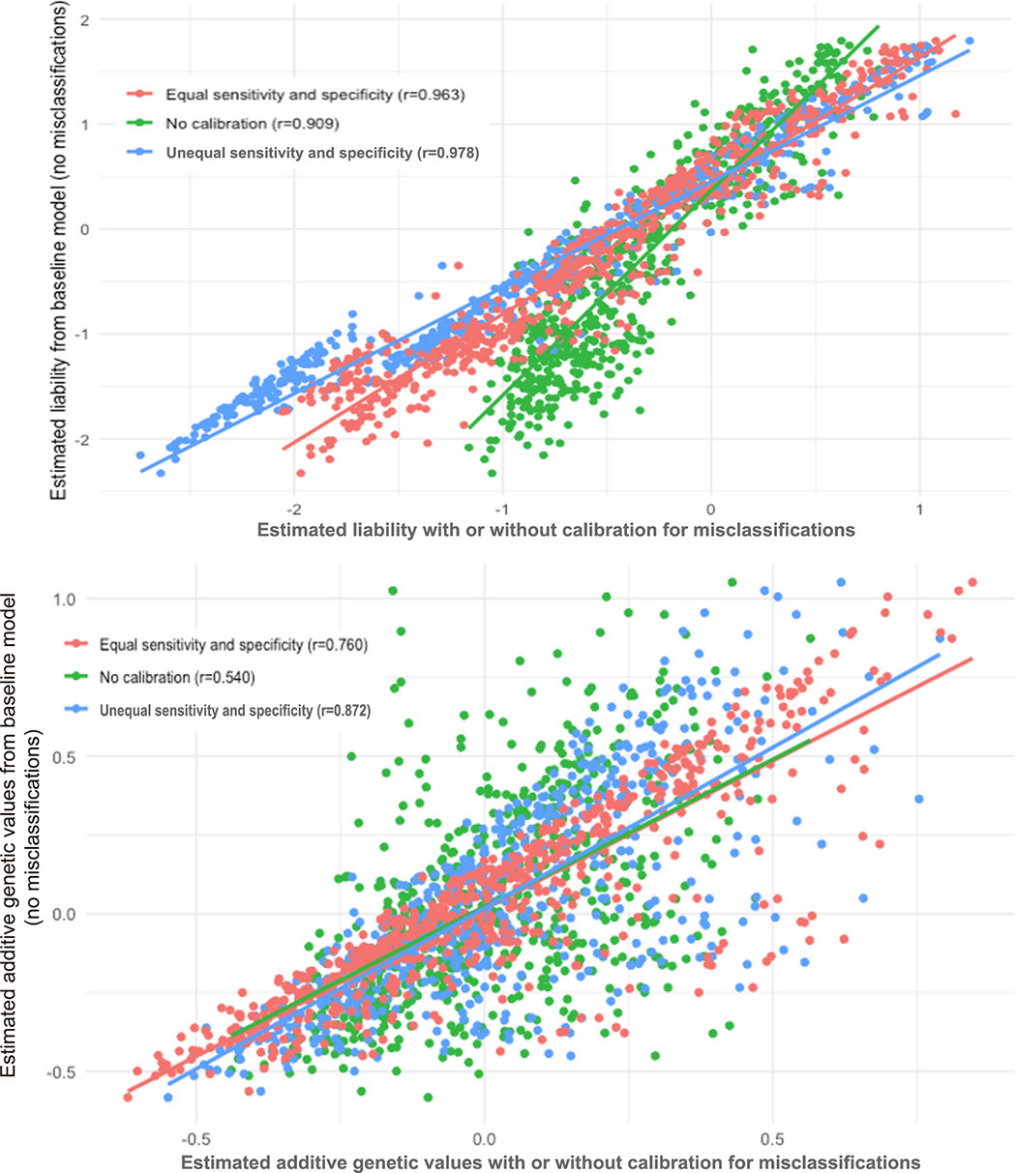
Comparing estimated liabilities (upper) and additive genetic values between a baseline model without misclassifications and 3 other models with nonzero misclassifications. No calibration = a threshold model without calibrating misclassifications; equal sensitivity and specificity = a threshold model assuming $\pi_{0|0}=\pi_{1|1}=0.85;$ unequal sensitivity and specificity = a threshold model assuming $\pi_{0|0}=0.8$ and $\pi_{1|1}=0.9.$

In summary, handling phenotypic errors requires proper calibration and modeling methods, often through pilot or independent studies. Theories related to genetic evaluation and analyses of discrete traits offer insights into their potential impact. This study does not provide a panacea for dealing with phenotypic errors in all scenarios, but it represents a preliminary effort to underscore the importance of recognizing and addressing these often-overlooked issues. For high-throughput phenotypes in particular, implementing validation subsets where a portion of the data is cross-referenced against high-accuracy measurements to estimate phenotyping errors would be a good idea. Additionally, hierarchical models or Bayesian approaches may be advantageous when working with herd-test data of varying reliability because they allow for differential weighting of phenotypic records based on their known or estimated accuracy.
